# Correlation between ambulatory blood pressure indices and sex hormone imbalance in hypertensive women with different menopausal durations: a cross-sectional study

**DOI:** 10.1186/s40001-025-03797-5

**Published:** 2026-01-19

**Authors:** Sijie Zhang, Heng Yu, Chenyang Jing, Wei Liang, Lei Chen, Yurui Lin, Ruxuan Li, Jing Yu, Ningyin Li

**Affiliations:** 1 Department of Cardiology, The Second Hospital and Clinical Medical School, Lanzhou University, No. 82 Cuiyingmen, Lanzhou, 730000 Gansu People’s Republic of China; 2 Department of Cardiology, Shanghai Public Health Clinical Center, Fudan University, No. 2901 Caolang Road, Jinshan District, Shanghai, 201508 People’s Republic of China

**Keywords:** Postmenopause, Menopausal duration, Hypertension, Sex hormone, Nocturnal blood pressure

## Abstract

**Background:**

The prevalence of hypertension in postmenopausal women increases significantly compared to premenopausal women and gradually catches up with, or even exceeds, that of their male counterparts. Imbalanced levels of sex hormones play a critical role in the elevation of blood pressure (BP) observed in this population. Furthermore, blunted nocturnal BP decline is a key factor contributing to their increased cardiovascular risk. This study compared ambulatory blood pressure (ABP) values between female hypertensive patients with different menopausal durations and matched male patients, aiming to explore the correlation between sex hormone imbalance and ABP values.

**Methods:**

This cross-sectional study included 210 postmenopausal women and 210 matched male patients. The male patients were matched to hypertensive female patients with varying menopausal durations on the basis of variables such as age, body mass index (BMI, kg/m^2^), hypertension duration, antihypertensive medication use and metabolism-related indicators. Clinical characteristics, Ambulatory Blood Pressure Monitoring (ABPM), and sex hormone levels were evaluated. Quantitative data were analyzed using one-way analysis of variance and multiple regression analysis.

**Results:**

Compared with matched men, in women with hypertension within 5 years after menopause, the T/PRL–E_2_ was associated with nighttime SBP. In those with menopausal durations of 6–10 years, the FSH/T ratio is associated with the absence of significant differences in 24-h SBP and daytime SBP relative to matched men, while the PRL/FSH ratio is negatively correlated with nighttime SBP in this group. In hypertensive women with menopausal durations of 11–15 years, the PRL-E_2_/T ratio is linked to 24-h, daytime and nighttime SBPs. In contrast, correlations involving diastolic blood pressure (DBP) indices displayed a distinct pattern. The FSH/PRL ratio shows a protective association with all mean DBP parameters in hypertensive women with menopausal durations less than 5 years and 11–15 years, whereas the T/P ratio is positively associated with those with 6–10 years of menopause.

**Conclusions:**

There was a significant correlation between sex hormone levels and ABP parameters, particularly nocturnal SBP, in women with different menopausal durations.

*Trial registration*: This study was retrospectively registered on ClinicalTrials.gov (NCT03451747) on February 18, 2018.

## Introduction

Hypertension is possibly the most powerful risk factor associated with the mortality and morbidity of cardiovascular disease (CVD) [1]. Multiple studies have shown that premenopausal women generally have lower BP levels than age-matched men do [2, 3]. Although SBP increases with age in both men and women, in postmenopausal women, the SBP increases significantly and may even exceed that of age-matched men [4, 5]. Rapid increases in BP after menopause may be associated with physiological changes specific to postmenopausal women, particularly altered sex hormone balance resulting from ovarian failure [6–8]. Therefore, correlation studies on postmenopausal hypertension hold important clinical significance for the prevention and treatment of CVD [9].

Under the regulation of circadian rhythms, the activity of the sympathetic nervous system is physiologically inhibited night. However, the sympathetic nervous system enhances vagus nerve-mediated parasympathetic activity, collectively leading to a physiological lowering of nocturnal BP, known as the “nocturnal BP dipping” phenomenon [10]. Non-dipping nocturnal BP, manifesting as blunted nocturnal BP decline [11], has a complex pathophysiology involving multiple mechanisms [12]: (1) genetic factors; (2) disruptions in the sleep‒activity cycle; (3) autonomic nervous system dysfunction; (4) alterations in water and sodium regulation; and (5) inflammation [13]. Compared with daytime BP elevation, blunted nocturnal BP decline induces more severe hypertension-mediated organ damage (HMOD) [14, 15]. Moreover, accumulating evidence has established that nocturnal BP offers substantially greater predictive value for cardiovascular events than office BP and other parameters derived from ABPM, including 24-h BP and daytime BP [16]. Thus, the regulation of nocturnal BP in postmenopausal women is crucial for preventing and managing HMOD [17].

Our team confirmed that postmenopausal women exhibit blunted or absent nocturnal BP decline, and this phenomenon is associated with sex hormone imbalance [18]. Specifically, a marked decrease in the level of estradiol (E_2_) disrupts autonomic nervous function and causes vascular endothelial dysfunction, impairing nocturnal BP decline [19]. Notably, such blunted nocturnal BP decline is also regarded as a strong predictor of an increased risk of CVD [15].

Sex hormones include follicle-stimulating hormone (FSH), luteinizing hormone (LH), E_2_, testosterone (T), prolactin (PRL), and progesterone (P) [20]. These hormones exhibit both autonomous activity and mutual regulation [21]. The secretion of ovarian hormones, such as E_2_, T, and P, is regulated by the hypothalamic‒pituitary‒ovarian (HPO) axis, which maintains the dynamic balance of the sex hormone environment through the interaction of hormones such as gonadotropin-releasing hormone, LH, and FSH [21]. While numerous studies have characterized how specific sex hormones, such as E_2_ and T, regulate BP [22, 23], recent studies highlight the limitations in the regulation of BP [24]. Increasingly, research suggests that BP regulation in postmenopausal women is influenced by the integrated balance and interactions among multiple sex hormones, rather than the level of any single hormone [24]. Differences in sex hormone balance across distinct menopausal stages in women may also lead to distinct variations in vascular function and BP regulation [24].

On the basis of the internationally recognized STRAW + 10 staging system for reproductive aging, this study classified postmenopausal women into three distinct groups (within 5 years from menopause, 6–10 years after menopause, and 11–15 years since menopause) [25]. Moreover, this study aimed to control for the confounding effects of aging and other factors while exploring sex differences in BP between postmenopausal women and age-matched men. For this purpose, male patients were matched to hypertensive female patients with varying menopausal durations on the basis of variables such as age, BMI, hypertension duration, antihypertensive medication use and metabolism-related indicators.

This study aimed to explore the association between the sex hormone imbalance and ABP values in hypertensive women with different menopausal durations. This was achieved by comparing their ABP values with those of matched male patients.

## Methods

### Study design and participants

This was a retrospective cross-sectional study. The minimum sample size required for each group in this study was calculated by the independent sample frequency test formula N = ${\left[\frac{2\left({u}_{\alpha }+{u}_{\upbeta }\right)\sigma }{\updelta }\right]}^{2}$. For one side, α = 0.05 and β = 0.10 were taken; through the corresponding table, we obtained uα = 1.96 and uβ = 1.28. In accordance with the results of previous studies, the maximum value of σ (σ = 8.1) and the minimum value of δ (δ = 3.7) were incorporated into the formula N = ${\left(\frac{2\times \left(1.96+1.28\right)\times 8.1}{3.7}\right)}^{2}$. A total of 420 hypertensive patients whose ABP was evaluated between November 2023 and May 2024 were enrolled. These participants, including 210 postmenopausal hypertensive females and 210 age-matched hypertensive males, were from the hypertension center of Lanzhou University Second Hospital. The medical records of the patients were reviewed and analyzed retrospectively by trained researchers.

### Inclusion and exclusion criteria

All of the participants met the following inclusion criteria: (1) were aged between 45 and 60 years; (2) had essential hypertension; and 3) had experienced natural menopause (for female patients). The definition of natural menopause was a lack of menstruation for 12 months.

Participants with untreated hypertension, secondary hypertension, white-coat hypertension, diabetes mellitus, thyroid disease, congestive heart failure, myocardial infarction, coronary artery diseases, atrial fibrillation, heart failure, severe valvular heart disease, cerebrovascular disease, stroke, liver dysfunction, renal insufficiency, peripheral vascular disease, autoimmune diseases, cancer, or other chronic diseases were excluded from the study. And any history of menopausal hormone therapy, including oral, transdermal, or other formulations, was an exclusion criterion.

In this study, male patients were matched to hypertensive female patients with varying menopausal durations on the basis of variables such as age, BMI, hypertension duration, antihypertensive medication use and metabolism-related indicators. Group a included hypertensive women with 5 years since menopause (N = 70), and Group a' included the men matched to the women in Group a (N = 70); Group b included hypertensive women with 6–10 years since menopause (N = 70), and Group b' included the men matched to the women in Group b (N = 70); and Group c included hypertensive women with 11–15 years since menopause (N = 70), and Group c' included the men matched to the women in Group c (N = 70).

### Data collection

The demographic information, clinical characteristics, and laboratory parameters, including age, sex, medication history, BMI, serum uric acid (UA) level, creatinine (Cr) level, fasting plasma glucose (FPG) level, lipid profile, and sex hormone levels, were collected from the patients’ clinical records.

### Laboratory blood tests

Blood samples were collected from the antecubital vein of each participant in the morning between 08:00 and 09:30 am after an overnight fasting period of 12 h. All of the blood indicators were measured by an ELAN autoanalyzer (Eppendorf, Germany) at the Central Laboratory of Lanzhou University Second Hospital. The criteria of the National Standard Laboratory (a WHO collaboration center in Tehran) were met by the laboratory for quality control measures. A Beckman SYNCHRON CX7 Analyzer (Beckman, Fullerton, CA, USA) with Beckman reagent kits was used for all biochemical analyses. The impedance method was used to measure white blood cell (WBC), red blood cell (RBC) and blood platelet (PLT) counts. The hemoglobin (Hb) level was determined by spectrocolorimetry. The FPG level was determined by the glucose oxidase enzymatic method. The levels of total cholesterol (TC) and triglycerides (TGs) were measured by standard enzymatic methods. The high-density lipoprotein cholesterol (HDL-C) level was determined by the dextran sulfate‒magnesium chloride precipitation method. The Friedewald formula was used to determine the level of low-density lipoprotein cholesterol (LDL-C). Aspartate aminotransferase (AST), alanine aminotransferase (ALT), blood urea nitrogen (BUN), and UA and Cr levels were measured by an automatic biochemical analyzer. The levels of sex hormones, including FSH, LH, E2, T, PRL, and P, were determined by chemiluminescence, and an electrochemical luminescence analysis system (Roche, CobasE-601; Roche, Basel, Switzerland) was used for detection.

### ABPM

ABPM was performed in all participants using the MOBI-O-GRAPH PWA, Germany. An appropriately sized cuff was placed on the non-dominant upper arm of each subject. BP readings were automatically obtained at 30-min intervals during daytime hours and 60-min intervals at night. Participants were instructed to adhere to their usual daily activities and prescribed medications throughout the monitoring period, which was conducted on regular working days. A valid ABPM recording was defined as one containing at least 80% of the scheduled measurements.

### Statistical analysis

All the data in this study were statistically analyzed by SPSS 27.0 statistical software (IBM, Armonk, New York, USA). The normality of continuous variables was evaluated through the Shapiro‒Wilk test, whereas the homogeneity of variance was assessed through Levene's test. Continuous variables with a normal distribution and homogeneity of variance are presented as the mean and standard deviation (SD), whereas continuous variables that did not conform to a normal distribution and/or had unequal variances are presented as the median (P25, P75). Intergroup analyses were conducted using one-way ANOVA (assuming homogeneity of variance) or the Kruskal‒Wallis test (for nonparametric distributions). Categorical variables were evaluated through chi-square tests. Multiple linear regression analysis was used to assess the associations between sex hormone levels and ABP parameters. A two-tailed P value less than 0.05 was considered to indicate statistical significance.

## Results

### Clinical characteristics

In our study, 420 patients were enrolled, including 210 postmenopausal patients with hypertension and 210 matched male patients with hypertension. The main clinical characteristics of all the participants are shown in Table 1. Male patients were matched with postmenopausal patients with hypertension with varying menopausal durations on the basis of variables such as age, BMI, hypertension duration, antihypertensive medication use and metabolism-related indicators. The menopause duration differed significantly among the three postmenopausal groups (*P* < 0.05). The RBC count and Hb, Cr and UA levels were lower in women with hypertension with any menopausal duration than in the matched male patients with hypertension (*P* < 0.05). Women with hypertension in the 11–15 years since menopause group presented lower WBC counts than their matched male counterparts did (*P* < 0.05), and higher K level than the women with hypertension in the 6–10 years since menopause group (*P* < 0.05). The other parameters were not significantly different between the groups.

**Table 1:** Clinical characteristics of patient groups (n = 420)

Variables	Total	Group a	Group b	Group c	Group a'	Group b'	Group c'
	(N = 420)	(N = 70)	(N = 70)	(N = 70)	(N = 70)	(N = 70)	(N = 70)
Age (years)	53.10 ± 0.21	48.89 ± 2.82	53.09 ± 1.59^*^	58.01 ± 2.21^*+^	47.96 ± 2.24	52.93 ± 1.29^**^	57.76 ± 1.90^** ++^
BMI (kg/m^2^)	23.35 ± 2.72	23.59 ± 3.96	23.16 ± 2.37	23.27 ± 2.26	23.52 ± 2.50	23.08 ± 2.41	23.48 ± 2.51
Menopausal durations (year)	7.30 ± 4.26	2.45 ± 1.00	7.12 ± 1.27^*^	12.34 ± 1.52^*+^	NA	NA	NA
WBCs (× 10^9^/L)	6.31 ± 1.54	6.11 ± 1.50	6.06 ± 1.52	6.06 ± 1.78	6.61 ± 1.55	6.42 ± 1.46	6.61 ± 1.36^**$**^
RBCs (× 10^12^/L)	4.81 ± 0.54	4.58 ± 0.53	4.59 ± 0.46	4.60 ± 0.44	5.05 ± 0.54^*^	5.08 ± 0.47^**+**^	4.96 ± 0.53^**$**^
Hb (g/L)	145.55 ± 17.04	136.51 ± 19.49	136.06 ± 13.97	139.59 ± 13.41	152.69 ± 14.09^*^	156.49 ± 13.44^**+**^	151.94 ± 14.32^**$**^
PLTs (× 10^9^/L)	206.89 ± 53.89	216.47 ± 55.17	205.00 ± 52.50	199.93 ± 53.70	209.50 ± 53.71	208.66 ± 54.34	201.77 ± 54.15
RDWSD (fL)	44.05 ± 3.35	43.37 ± 3.82	44.11 ± 4.00	43.50 ± 3.01	44.16 ± 2.88	44.81 ± 3.05	44.37 ± 3.08
RDWCV (fL)	13.07 ± 0.94	13.04 ± 1.00	13.24 ± 1.04	12.96 ± 0.92	12.97 ± 0.89	13.06 ± 0.95	13.13 ± 0.84
K (mmol/L)	3.78 ± 0.36	3.76 ± 0.35	3.69 ± 0.38	3.84 ± 0.37^**+**^	3.78 ± 0.36	3.80 ± 0.35	3.79 ± 0.34
Na (mmol/L)	140.52 ± 3.02	140.29 ± 3.34	141.03 ± 2.61	141.13 ± 2.69	139.70 ± 3.09	140.58 ± 2.93	140.40 ± 3.26
Cr (μmmol/L)	73.38 ± 21.98	67.62 ± 21.62	67.70 ± 23.76	67.02 ± 22.05	80.38 ± 19.14^*^	80.51 ± 22.40^**+**^	77.07 ± 17.99^**$**^
BUN (mmol/L)	5.58 ± 1.61	5.24 ± 1.46	5.42 ± 1.66	5.74 ± 1.60	5.69 ± 1.68	5.73 ± 1.53	5.69 ± 1.74
UA (μmmol/L)	332.73 ± 89.52	300.80 ± 73.63	295.17 ± 71.25	275.93 ± 88.52	366.16 ± 77.48^*^	375.41 ± 83.93^**+**^	382.91 ± 77.53^**$**^
TGs (mmol/L)	1.69 ± 0.63	1.63 ± 0.57	1.61 ± 0.63	1.70 ± 0.70	1.70 ± 0.61	1.80 ± 0.58	1.71 ± 0.68
TC (mmol/L)	4.15 ± 0.89	4.22 ± 0.81	4.12 ± 0.93	4.37 ± 0.88	3.94 ± 0.86	4.17 ± 0.95	4.08 ± 0.87
HDL (mmol/L)	1.18 ± 0.27	1.21 ± 0.29	1.20 ± 0.22	1.24 ± 0.26	1.13 ± 0.25	1.15 ± 0.27	1.17 ± 0.31
LDL (mmol/L)	2.44 ± 0.77	2.55 ± 0.70	2.32 ± 0.74	2.57 ± 0.82	2.39 ± 0.72	2.50 ± 0.82	2.32 ± 0.78
ALT (U/L)	28.58 ± 10.11	26.06 ± 9.19	29.17 ± 9.05	26.80 ± 11.02	28.43 ± 10.64	31.49 ± 9.39	29.53 ± 10.56
AST (U/L)	26.47 ± 8.14	24.67 ± 9.22	26.34 ± 8.51	25.87 ± 6.55	27.29 ± 8.42	28.09 ± 7.35	26.57 ± 8.39

Statistical significance was defined as *P* < 0.05

^*^P < 0.05 compared with Group a

^**^P < 0.05 compared with Group a′

^+^*P* < 0.05 compared with Group b

^++^*P* < 0.05 compared with Group b′

^$^*P* < 0.05 compared with Group c

^$$^*P* < 0.05 compared with Group c′

### Sex hormone indices

Within the same sex group, intragroup comparisons revealed that the levels of P and the PRL-E_2_/T ratio in women with hypertension in the 11–15 years since menopause group were significantly lower than those in women with hypertension in the 5 years since menopause group and those in the 6–10 years since menopause group (Table 2, *P* < 0.05). Similarly, the FSH level and T level in women with hypertension in the 11–15 years since menopause group was higher than that in women with hypertension in the 5 years since menopause group (*P* < 0.05). There were no significant differences in the other parameters among the groups.

**Table 2:** Sex hormone indices of patient groups (n = 420)

Variables	Total	Group a	Group b	Group c	Group a′	Group b′	Group c′
	(N = 420)	(N = 70)	(N = 70)	(N = 70)	(N = 70)	(N = 70)	(N = 70)
PRL (ng/ml)	10.14 ± 3.54	11.05 ± 3.28	10.90 ± 4.17	9.96 ± 3.41	9.85 ± 2.82^*^	9.17 ± 3.56^**+**^	9.89 ± 3.60
FSH (mIU/ml)	34.00 ± 30.23	56.38 ± 18.94	61.43 ± 25.29	62.05 ± 19.90^*^	7.89 ± 3.24^*^	7.99 ± 3.44^**+**^	8.25 ± 3.35^**$**^
LH (mIU/ml)	16.89 ± 13.63	29.72 ± 9.42	28.62 ± 8.61	28.31 ± 8.78	4.65 ± 1.99^*^	5.12 ± 1.84^**+**^	4.92 ± 1.98^**$**^
E_2_ (pg/ml)	27.25 ± 9.90	29.63 ± 9.49	28.55 ± 8.57	28.47 ± 8.76	24.43 ± 10.53^*^	26.76 ± 9.49	25.64 ± 11.56
T (ng/dl)	177.16 ± 176.03	22.67 ± 9.02	18.41 ± 6.83	16.67 ± 6.75^*^	326.91 ± 95.70^*^	333.02 ± 113.13^**+**^	345.25 ± 118.59^**$**^
P (ng/ml)	0.49 ± 0.19	0.53 ± 0.16	0.48 ± 0.16	0.41 ± 0.17^*+^	0.50 ± 0.21	0.51 ± 0.21	0.53 ± 0.21^**$**^
FSH/T (mIU/ng)	0.26(0.24,3.42)	2.51(1.78,4.10)	3.50(2.14,5.27)	4.01(2.96,5.10)	0.03(0.02,0.03)^*^	0.02(0.02,0.03)^**+**^	0.02(0.02,0.03)^**$**^
FSH/PRL (mIU/ng)	1.78(0.89,6.27)	5.54(3.76,3.70)	6.44(4.30,7.33)	6.66(5.07,8.09)	0.79(0.63,1.12)^*^	0.94(0.68,1.25)^**+**^	0.91(0.55,1.22)^**$**^
T/P (10^2^)	161.74 (40.77,661.88)	42.78 (29.65,57.75)	40.19 (23.67,60.33)	38.18 (25.81,63.16)	630.51 (442.93,938.18)^*^	654.29 (441.04,1039.30)^**+**^	681.75 (448.78,1070.32)^**$**^
PRL/FSH (ng/mIU)	0.56 (0.16,1.12)	0.18 (0.14,0.27)	0.16 (0.14,0.23)	0.15 (0.12,0.20)	1.26 (0.90,1.60)^*^	1.06 (0.80,1.46)^**+**^	1.10 (0.82,1.83)^**$**^
PRL-E_2_/T (ng/ml)	9.01 (6.51,11.32)	9.59 (7.05,11.35)	9.11 (5.84,12.21)	7.30 (5.26,10.25)^*+^	9.34 (8.17,11.53)^*^	8.58 (6.22,11.59)^**+**^	9.25 (7.38,13.34)^**$**^
T/PRL-E_2_ (ng/ml)	-11.98 (-26.33,10.41)	-27.42 (-34.59,-18.71)	-26.24 (-31.05,-20.68)	-25.92 (-33.18,-20.88)	8.59 (0.52,18.70)^*^	11.90 (0.31,29.84)^**+**^	10.03 (0.48,21.47)^**$**^

Statistical significance was defined as *P* < 0.05

^*^P < 0.05 compared with Group a

^**+**^*P* < 0.05 compared with Group b

^**$**^*P* < 0.05 compared with Group c

However, comparisons between postmenopausal women with hypertension and their matched male counterparts revealed that women with hypertension in the 5 years since menopause group had a higher level of E_2_ than their matched male counterparts did (*P* < 0.05). The levels of FSH, LH, the FSH/T ratio, the FSH/PRL ratio and the E_2_/T ratio in all the postmenopausal women were significantly greater than those in the matched male patients (*P* < 0.05). Compared with the matched male patients, all postmenopausal female patients had lower T levels, T/P ratios, PRL/FSH ratios and T/PRL ratio-E_2_ levels (*P* < 0.05). Additionally, the levels of PRL and the PRL-E_2_/T ratio in women with hypertension in the 5 years since menopause group and those in the 6–10 years since menopause group were greater than those in their matched male counterparts (*P* < 0.05). However, the levels of the PRL-E_2_/T ratio and P in women with hypertension in the 11–15 years since menopause group were lower than those in their matched male counterparts (*P* < 0.05).

### ABP characteristics

Women with hypertension in the 5 years since menopause group had 24-h SBP and daytime SBP significantly lower than those in their matched male counterparts did (Table 3, *P* < 0.05). However, these differences were not observed in the other postmenopausal groups (*P* > 0.05). Compared with their male counterparts, all the postmenopausal women presented lower 24-h DBP, daytime DBP, and nighttime DBP (*P* < 0.05). According to the intragroup comparisons, postmenopausal women in the 6–10 years since menopause group presented a lower morning SBP than did those in the 5 years since menopause group (*P* < 0.05).

**Table 3:** Basic BP and pulse rate indices of patient groups (n = 420)

Variables	Total	Group a	Group b	Group c	Group a′	Group b′	Group c′
	(N = 420)	(N = 70)	(N = 70)	(N = 70)	(N = 70)	(N = 70)	(N = 70)
24-h SBP (mmHg)	133.35 ± 16.91	130.00 ± 18.84	131.80 ± 15.40	134.67 ± 16.86	135.64 ± 16.57^*^	133.80 ± 16.92	134.17 ± 16.64
24-h DBP (mmHg)	81.01 ± 10.78	78.24 ± 10.23	80.19 ± 8.93	78.01 ± 10.16	83.80 ± 10.95^*^	83.79 ± 12.01^**+**^	82.04 ± 10.92^**$**^
daytime SBP (mmHg)	135.83 ± 17.17	131.89 ± 19.38	134.77 ± 15.61	137.26 ± 17.51	138.19 ± 16.83^*^	136.27 ± 17.09	136.60 ± 16.23
daytime DBP (mmHg)	82.81 ± 11.39	79.40 ± 11.58	81.79 ± 9.71	80.13 ± 10.60	85.84 ± 11.32^*^	85.59 ± 12.36^**+**^	84.14 ± 11.30^**$**^
nighttime SBP (mmHg)	126.91 ± 18.84	124.89 ± 19.39	124.60 ± 18.30	129.41 ± 18.83	127.67 ± 17.98	127.30 ± 18.99	127.60 ± 19.71
nighttime DBP (mmHg)	77.41 ± 11.21	75.60 ± 10.63	76.03 ± 9.93	74.16 ± 11.38	80.09 ± 10.37^*^	80.00 ± 12.03^**+**^	78.59 ± 11.75^**$**^
morning SBP (mmHg)	136.61 ± 18.79	138.09 ± 22.63	131.89 ± 15.04^*^	133.56 ± 17.47	140.83 ± 17.89	136.10 ± 19.44	139.23 ± 18.50
morning DBP (mmHg)	83.87 ± 12.63	85.04 ± 14.97	81.13 ± 11.58	81.91 ± 10.74	87.11 ± 11.72	83.80 ± 13.52	84.23 ± 12.28
24-h mean PR (bpm)	71.43 ± 8.17	71.30 ± 8.14	72.19 ± 7.96	69.54 ± 10.49	72.83 ± 7.60	71.04 ± 6.27	71.66 ± 7.91
daytime mean PR (bpm)	75.46 ± 8.21	76.29 ± 8.59	75.89 ± 7.91	73.57 ± 10.13	76.86 ± 7.03	75.39 ± 7.21	74.77 ± 7.90
nighttime mean PR (bpm)	67.35 ± 8.38	67.64 ± 8.88	68.31 ± 7.34	65.74 ± 10.68	67.86 ± 7.44	66.86 ± 7.40	67.66 ± 8.06

Statistical significance was defined as *P* < 0.05

^*^P < 0.05 compared with Group a

^**+**^*P* < 0.05 compared with Group b

^**$**^*P* < 0.05 compared with Group c

Women with hypertension in the 5 years since menopause group had significantly lower 24-h SBP loads and 24-h DBP loads than their matched male counterparts did (Table 4, *P* < 0.05). Additionally, female patients in the 11–15 years since menopause group had a lower 24-h DBP loads, daytime DBP load and nighttime DBP load than male patients did (*P* < 0.05).

**Table 4:** BP load indices of patient groups (n = 420)

Variables	Total	Group a	Group b	Group c	Group a′	Group b′	Group c′
	(N = 420)	(N = 70)	(N = 70)	(N = 70)	(N = 70)	(N = 70)	(N = 70)
24-h SBP load (%)	0.53 ± 0.16	0.51 ± 0.16	0.50 ± 0.16	0.52 ± 0.19	0.57 ± 0.15^*^	0.55 ± 0.13	0.54 ± 0.14
24-h DBP load (%)	0.52 ± 0.18	0.50 ± 0.19	0.51 ± 0.18	0.45 ± 0.19	0.57 ± 0.17^*^	0.57 ± 0.17	0.52 ± 0.18^**$**^
Daytime SBP load (%)	0.51 ± 0.17	0.48 ± 0.18	0.49 ± 0.17	0.50 ± 0.20	0.54 ± 0.18	0.53 ± 0.15	0.52 ± 0.16
Daytime DBP load (%)	0.48 ± 0.20	0.47 ± 0.20	0.47 ± 0.20	0.41 ± 0.21	0.52 ± 0.18	0.53 ± 0.19	0.48 ± 0.19^**$**^
Nighttime SBP load (%)	0.57 ± 0.17	0.58 ± 0.18	0.54 ± 0.18	0.55 ± 0.20	0.57 ± 0.17	0.57 ± 0.16	0.58 ± 0.16
Nighttime DBP load (%)	0.60 ± 0.18	0.61 ± 0.19	0.62 ± 0.17	0.56 ± 0.21	0.61 ± 0.16	0.60 ± 0.16	0.63 ± 0.18^**$**^

Statistical significance was defined as *P* < 0.05

^*^P < 0.05 compared with Group a

^**+**^*P* < 0.05 compared with Group b

^**$**^*P* < 0.05 compared with Group c

Most BP variation and SD indices were not significantly different among the groups (Table 5, *P* > 0.05).

**Table 5:** BP Variation and SD Indices of Patient Groups (n = 420)

Variables	Total	Group a	Group b	Group c	Group a′	Group b′	Group c′
	(N = 420)	(N = 70)	(N = 70)	(N = 70)	(N = 70)	(N = 70)	(N = 70)
24-h SBP variation (%)	0.13 ± 0.03	0.12 ± 0.03	0.13 ± 0.03	0.13 ± 0.03	0.12 ± 0.03	0.13 ± 0.03	0.13 ± 0.02
24-h DBP variation (%)	0.14 ± 0.03	0.13 ± 0.03	0.14 ± 0.03	0.14 ± 0.03	0.13 ± 0.04	0.14 ± 0.03	0.14 ± 0.03
Daytime SBP variation (%)	0.13 ± 0.04	0.12 ± 0.04	0.13 ± 0.04	0.14 ± 0.03	0.13 ± 0.04	0.13 ± 0.04	0.14 ± 0.03
Daytime DBP variation (%)	0.14 ± 0.04	0.13 ± 0.04	0.14 ± 0.04	0.14 ± 0.04	0.14 ± 0.04	0.14 ± 0.04	0.15 ± 0.03
Nighttime SBP variation (%)	0.11 ± 0.03	0.11 ± 0.03	0.10 ± 0.03	0.11 ± 0.03^**+**^	0.10 ± 0.03	0.10 ± 0.03	0.11 ± 0.03
Nighttime DBP variation (%)	0.12 ± 0.04	0.12 ± 0.04	0.12 ± 0.03	0.13 ± 0.04	0.12 ± 0.05	0.13 ± 0.04	0.13 ± 0.04
24-h SBP SD (mmHg)	12.84 ± 4.16	12.41 ± 3.85	12.76 ± 4.66	12.59 ± 4.51	13.76 ± 4.22	12.84 ± 3.40	12.69 ± 4.21
24-h DBP SD (mmHg)	12.92 ± 3.88	13.20 ± 2.99	13.21 ± 3.78	12.17 ± 4.33	12.94 ± 3.92	13.29 ± 4.06	12.69 ± 4.09
Daytime SBP SD (mmHg)	15.85 ± 4.37	15.67 ± 4.07	15.46 ± 4.86	16.54 ± 5.50	15.50 ± 3.89	15.81 ± 4.11	16.13 ± 3.54
Daytime DBP SD (mmHg)	10.92 ± 3.15	10.67 ± 3.24	10.47 ± 3.50	10.40 ± 3.61	11.54 ± 2.87	11.16 ± 2.99	11.29 ± 2.47
Nighttime SBP SD (mmHg)	16.89 ± 5.56	16.01 ± 5.36	16.19 ± 5.93	17.79 ± 5.14	17.21 ± 5.67	16.70 ± 6.02	17.44 ± 5.16
Nighttime DBP SD (mmHg)	11.34 ± 3.83	11.40 ± 3.70	10.24 ± 4.65	11.40 ± 2.96	11.47 ± 4.25	11.50 ± 3.39	12.03 ± 3.72
Morning SBP SD (mmHg)	14.14 ± 5.32	14.16 ± 4.39	13.09 ± 4.78	14.57 ± 6.49	14.41 ± 5.27	14.37 ± 5.58	14.26 ± 5.22
Morning DBP SD (mmHg)	11.06 ± 4.10	10.74 ± 4.57	10.80 ± 4.21	10.34 ± 4.52	11.77 ± 3.96	11.53 ± 3.82	11.20 ± 3.34
Morning SBP SD (mmHg)	12.84 ± 4.16	12.41 ± 3.85	12.76 ± 4.66	12.59 ± 4.51	13.76 ± 4.22	12.84 ± 3.40	12.69 ± 4.21
Morning DBP SD (mmHg)	12.92 ± 3.88	13.20 ± 2.99	13.21 ± 3.78	12.17 ± 4.33	12.94 ± 3.92	13.29 ± 4.06	12.69 ± 4.09

Statistical significance was defined as *P* < 0.05

^*^P < 0.05 compared with Group a

^**+**^*P* < 0.05 compared with Group b

^**$**^*P* < 0.05 compared with Group c

### Correlation analysis

**Fig. 1:**
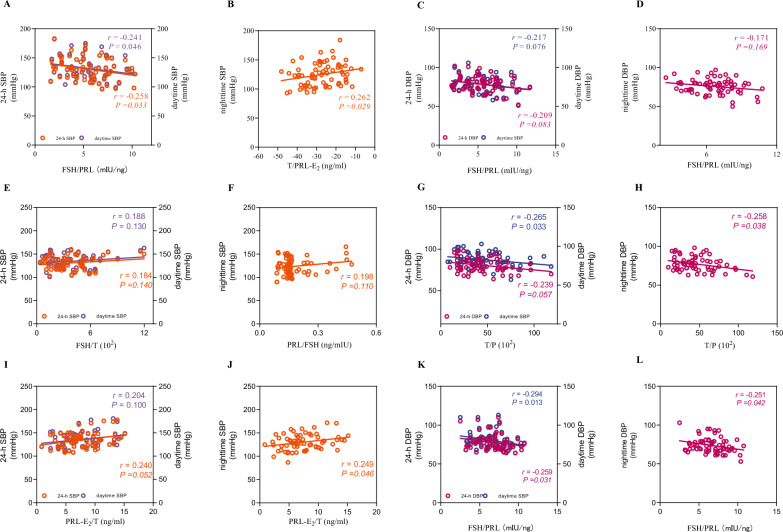
Correlations between sex hormones and ABP parameters in Group a–c. **A** shows the correlations between the FSH/PRL ratio and 24-h SBP, as well as daytime SBP in Group a. **B** shows the correlation between the T/PRL ratio-E_2_ and nighttime SBP in Group a. **C** shows the correlations between the FSH/PRL ratio and 24-h DBP, as well as daytime DBP in Group a. **D** shows the correlation between the FSH/PRL ratio and nighttime DBP in Group a. **E** shows the correlations between the FSH/T ratio and 24-h SBP, as well as daytime SBP in Group b. **F** shows the correlations between the PRL/FSH ratio and nighttime SBP in Group b. **G** shows the correlations between the T/P ratio and 24-h DBP, as well as daytime DBP in Group b. **H** shows the correlations between the T/P ratio and nighttime DBP in Group b. **I** shows the correlations between the PRL-E_2_/T ratio and 24-h SBP, as well as daytime SBP in Group c. **J** shows the correlations between the PRL-E_2_/T ratio and nighttime SBP in Group c. **K** shows the correlations between the FSH/PRL ratio and 24-h DBP, as well as daytime DBP in Group c. **L** shows the correlation between the FSH/PRL ratio and nighttime DBP in Group c

Spearman's rank correlation analysis was employed in this study to assess the relationships between variables (Fig. 1).

In hypertensive women within 5 years post menopause, the FSH/PRL ratio was significantly negatively correlated with 24-h SBP (r = −0.258, *P* = 0.033) and daytime SBP (r = −0.241, *P* = 0.046). Furthermore, it showed consistent inverse associations with 24-h (r = −0.209, *P* = 0.083), daytime (r = −0.217, *P* = 0.076), and nighttime DBP (r = −0.171, *P* = 0.169), although these correlations did not reach statistical significance. A positive correlation was observed between the T/PRL-E_2_ ratio and nighttime SBP in this group (r = 0.262, *P* = 0.029).

In women with hypertension in the 5–10 years since menopause group, the FSH/T ratio showed a positive numerical trend with both 24-h SBP (r = 0.184, *P* = 0.140) and daytime SBP (r = 0.188, *P* = 0.130). Similarly, the PRL/FSH ratio showed a positive trend with nighttime SBP (r = 0.195, *P* = 0.110). None of these associations reached statistical significance. The T/P ratio was inversely associated with daytime DBP (r = −0.265, *P* = 0.033), nighttime DBP (r = −0.258, *P* = 0.038), and 24-h DBP (r = −0.239, *P* = 0.057).

In women with hypertension in the 11–15 years since menopause group, the PRL-E_2_/T ratio was significantly positively correlated with nighttime SBP (r = 0.249, *P* = 0.046). Positive associations with 24-h SBP (r = 0.240, *P* = 0.052) and daytime SBP (r = 0.204, *P* = 0.100) were also observed, although these did not reach statistical significance. A negative correlation was observed between the FSH/PRL ratio and 24-h (r = −0.259, *P* = 0.031), daytime (r = −0.294, *P* = 0.013), and nighttime DBP (r = −0.251, *P* = 0.042) in this group.

### Multiple linear regression

Multivariate linear regression analysis indicated that variations in the sex hormone environments across different postmenopausal stages were associated with the observed differences in ABP between postmenopausal women and matched male patients with hypertension (Table 6, 7, 8). Confounding variables were adjusted for in the analysis, such as the red blood cell distribution width (RDWSD), red blood cell distribution width coefficient of variation (RDWCV), and Cr, UA, AST, ALT, and Na levels.

**Table 6:** Multivariate linear regression analysis of ABP in Group a

Variables	B	*P* value
24-h SBP		
FSH/PRL	− 2.01	0.033
24-h DBP		
FSH/PRL	− 1.13	0.028
Daytime SBP		
FSH/PRL	− 2.1	0.029
Daytime DBP		
FSH/PRL	− 1.19	0.039
Nighttime SBP		
T/PRL-E_2_	0.5	0.043
Nighttime DBP		
FSH/PRL	− 1.68	0.003

Statistical significance was defined as *P* < 0.05

**Table 7:** Multivariate linear regression analysis of ABP in Group b

Variables	B	*P* value
24-h SBP		
FSH/T	1.16	0.048
24-h DBP		
T/P	− 0.11	0.022
Daytime SBP		
FSH/T	1.27	0.033
Daytime DBP		
T/P	− 0.11	0.039
Nighttime SBP		
PRL/FSH	27.71	0.037
Nighttime DBP		
T/P	− 0.1	0.043

Statistical significance was defined as *P* < 0.05

**Table 8:** Multivariate linear regression analysis of ABP in Group c

Variables	B	*P* value
24-h SBP		
PRL-E_2_/T	1.56	0.021
24-h DBP		
FSH/PRL	− 1.63	0.016
Daytime SBP		
PRL-E_2_/T	1.94	0.036
Daytime DBP		
FSH/PRL	− 1.49	0.005
Nighttime SBP		
PRL-E_2_/T	2.34	0.002
Nighttime DBP		
FSH/PRL	− 1.8	0.018

Statistical significance was defined as *P* < 0.05

In women with hypertension in the 5 years since menopause group (Table 6), the FSH/PRL ratio was independently associated with 24-h SBP, 24-h DBP, daytime SBP, daytime DBP, and nighttime DBP (*P* < 0.05), whereas the T/PRL ratio-E_2_ level was independently associated with nighttime SBP (*P* < 0.05).

In women with hypertension in the 6–10 years since menopause group (Table 7), the FSH/T ratio was independently associated with 24-h and daytime SBP (*P* < 0.05), and the T/P ratio was independently associated with 24-h, daytime, and nighttime DBP (*P* < 0.05). The PRL/FSH ratio was independently associated with nighttime SBP (*P* < 0.05).

Among women with hypertension in the 11–15 years since menopause group (Table 8), the PRL-E_2_/T ratio was independently associated with 24-h, daytime, and nighttime SBP (*P* < 0.05). The FSH/PRL ratio was independently associated with the 24-h, daytime, and nighttime DBP (*P* < 0.05).

## Discussion

This cross-sectional study demonstrated that women with hypertension at different stages post-menopause exhibited distinct ABP profiles. The sex hormone environment was found to be associated with overall regulation of BP across these stages within the postmenopausal hypertensive population. Furthermore, key findings included a gradual narrowing of the SBP difference between postmenopausal women and matched men, and the observation that the impaired regulation of nocturnal SBP appears to constitute an early, pivotal step in the subsequent rise of overall SBP in postmenopausal women.

### Sex hormone environments and vascular function across postmenopausal stages

Perimenopause is marked by a pronounced decline in oocyte number and antral follicle count (AFC), accompanied by diminishing follicular function, which initiates early fluctuations in sex hormone levels [25]. As ovarian structure and function evolve after menopause, further alterations in the hormonal environment occur [26]. A hallmark of this transition is a sharp decline in E_2_, which not only reduces its cardioprotective effects but also activates the HPO axis negative feedback, leading to elevated FSH levels [22, 27]. To better understand the relationship between postmenopausal duration, sex hormones, and BP, we stratified women into three groups according to the STRAW + 10 staging system [25]. Group a (early post menopause: stages + 1b, + 1c) was characterized by very low AFC, rapidly rising FSH, and sharply declining—then stabilizing—E_2_ levels [25, 28, 29]. Group b (transitioning to late post menopause: stage + 1c to + 2) showed further hormonal alterations [25]. Group c (late post menopause: stage + 2) exhibited complete AFC depletion, stable reproductive endocrine function, and minimal hormone fluctuations [25, 29]. Although distinct hormonal shifts occur across these stages, data covering the full transition from + 1 to + 2 remain limited and merit further study [25].

A recent study using the STRAW + 10 criteria confirmed a relationship between menopausal status and arterial stiffness, noting a significant acceleration in aortic stiffening specifically in late post menopause (stage + 2), but not during perimenopause [30]. FMD has also been shown to gradually decline from pre-menopause through post menopause [31]. Collectively, this evidence suggests that stage-specific changes in ovarian function and sex hormones may differentially influence vascular function and BP regulation.

### Nocturnal SBP dysregulation: a key early feature of postmenopausal hypertension

Dysregulation of nocturnal SBP and blunted nocturnal SBP decline are common in postmenopausal women and are largely attributed to autonomic nervous system dysregulation and vascular dysfunction [32–35]. Although the precise mechanisms remain incompletely understood, postmenopausal hormonal changes, particularly the acute drop in E_2_, are strongly implicated [36]. E_2_ suppresses sympathetic activity [37], its reduction leads to increased sympathetic tone, which correlates positively with peripheral resistance. Moreover, unlike men, postmenopausal women lack a compensatory decrease in cardiac output to buffer rising peripheral resistance, further disrupting BP control [38]. Recent evidence also indicates that E_2_ deficiency inhibits the NRF2/GPX4 pathway, promoting endothelial ferroptosis and accelerating vascular dysfunction and atherosclerosis [39].

In contrast, the role of T in autonomic function and nocturnal BP in postmenopausal women remains underexplored. In our previous work, however, higher T levels were associated with elevated nocturnal SBP and arterial stiffness [18]. Other studies suggest that both FSH and PRL contribute to arterial stiffening and impaired endothelium-dependent vasodilation [40–44]. A case–control study identified PRL > 8.0 ng/mL as an independent risk factor for impaired nocturnal dipping in postmenopausal women [45]. The synergistic action of these hormones appears critical to the suppression of nocturnal BP decline.

A distinct pattern of sex-specific SBP convergence emerges among women with hypertension after menopause. Among women within 5 years of menopause, nocturnal SBP shows no significant difference from that of matched male patients (Table 3, *P* > 0.05). In contrast, the sex disparity in daytime and 24-h SBP diminishes more gradually, aligning only with prolonged postmenopausal duration (Table 3, *P* < 0.05). This differential timing points to a specific pathophysiology: the rising SBP in early postmenopausal women may not merely reflect impaired nocturnal dipping. Instead, it could signal an early, pivotal shift within the systemic SBP trajectory, a process fundamentally linked to the unique remodeling of the sex hormone environment during this transitional phase.

Physiologically, this period is defined by a marked endocrine shift. E_2_ levels fall precipitously, while the hormonal landscape for T becomes more nuanced [24]. Although direct ovarian secretion of T declines, enhanced peripheral conversion of adrenal androstenedione to T often results in circulating T levels that are only mildly reduced or remain stable [24]. PRL levels during this stage typically show minimal change or a slight decrease [46]. Recognizing that T and PRL can act as vascular risk modulators whereas E_2_ confers protection, we posited that their integrated balance, rather than any single hormone, might be more physiologically relevant to nocturnal hemodynamics [18, 37, 40]. To better capture the interplay of these hormones, we developed a composite index, the T/PRL–E_2_, integrating key regulators of nocturnal BP. This index quantifies the balance between risk factors (T and PRL) and a protective factor (E_2_). In women with hypertension within 5 years post menopause (early post menopause), this index was independently associated with the regulation of nocturnal SBP (Table 6, *P* < 0.05).

### Differential SBP links in late post menopause: 6–10 versus 11–15 years

The hormonal landscape undergoes a fundamental reconfiguration during the late post menopause. Progressive ovarian decline attenuates HPO axis feedback, precipitating a sustained rise in FSH [21]. Notably, direct ovarian contribution to circulating T becomes minimal [24]. The primary source of T shifts to the peripheral conversion of adrenal precursors, a process that yields circulating levels markedly lower than those observed in the premenopausal period [24]. This shift results in a state of significant hormonal disequilibrium relative to the reproductive years. Within this altered endocrine environment, the FSH/T ratio demonstrates particular salience. Its progressive increase throughout the menopausal transition captures the essence of these pathophysiological changes, the escalating drive of the HPO axis against a backdrop of diminishing gonadal steroidogenic output [24]. Consequently, this ratio may hold considerable utility for assessing derangements in sex hormone homeostasis in postmenopausal women in the future.

More importantly, both FSH and T have been associated with increased arterial stiffness [18, 42]. Furthermore, T may promote vasoconstriction via upregulation of endothelin-1 (ET-1) and suppression of prostacyclin (PGI_2_), which can contribute to impaired arterial function and elevated SBP [47]. Notably, prior studies have reported a positive correlation between the FSH/T ratio and aortic atherosclerotic plaque burden, systemic inflammation, and incident cardiovascular events [48]. In the present study, we newly identify that an elevated FSH/T ratio is significantly associated with increased 24-h and daytime SBP among women who are 6–10 years postmenopausal (Table 7, *P* < 0.05). These findings underscore the synergistic influence of FSH and T on SBP elevation and reflect the underlying hormonal dysregulation typical of this postmenopausal stage.

Women who are 11–15 years post menopause (fully in late stage) experience complete ovarian failure, representing the end of reproductive aging [25]. At this stage, the relationship between sex hormones and SBP acquires new features: E_2_ declines sharply [22], whereas T decreases more modestly, resulting in a reduced E_2_/T ratio [24, 49]. This imbalance attenuates cardio protection. Incorporating the adverse effect of PRL, we developed a new index, PRL–E_2_/T, which was found to be a common risk factor for 24-h, daytime, and nocturnal SBP at this stage. This suggests a “rebalanced” but risk-promoting hormonal environment with uniform SBP regulation across all periods.

### Differential DBP links in late post menopause: 6–10 versus 11–15 years

Elderly women with hypertension in late post menopause (mean age 66.09 ± 4.78 years) have significantly lower DBP than men [50]. Notably, low DBP is associated with increased cardiovascular risk only in women, underscoring the need for sex-specific DBP management [50]. Anatomical factors, such as shorter arterial tree and higher heart rate in women, promote earlier return of reflected pressure waves, contributing to lower risks of lifelong DBP compared with men [4, 50, 51]. In late postmenopausal women, increased aortic stiffness, along with reduced vascular elasticity, decreased aortic elastic recoil, and premature wave reflection, collectively contributes to lower DBP [21, 52]. Endothelial dysfunction, indicated by reduced brachial artery FMD, also contributes to lower DBP via increased arterial stiffness [53].

Further analysis revealed that the relationship between DBP and the hormonal environment also varied by postmenopausal stage. In women 6–10 years post menopause (transitioning to late stage), a higher T/P ratio was associated with lower DBP (Table 8, *P* < 0.05). We hypothesize that this association may be mediated through synergistic mechanisms in endothelial regulation. T has dual vascular effects. It promotes vasodilation via NO synthesis and improves vascular repair via endothelial progenitor cells [54–56], but may also cause vasoconstriction via ET-1 and PGI_2_ [47]. P enhances endothelium-dependent dilation via eNOS/NO activation, inhibits ET-1, stimulates PGI_2_ [57, 58], and reduces monocyte-endothelial adhesion [59]. During this transitional phase, T and P may synergistically enhance NO release, whereas P counteracts T-induced vasoconstriction, helping maintain vasomotor balance and protecting DBP [54–59]. However, these speculations lack direct mechanistic evidence, and their exact molecular mechanisms await further investigation.

In the fully late postmenopausal group (11–15 years since menopause), the imbalance between a marked elevation in FSH and the relative stability of PRL collectively drives dynamic changes in the FSH/PRL ratio. Compared with single hormones, this unique characteristic positions the ratio as a promising sensitive indicator for quantifying the progression of ovarian reserve exhaustion and the instability of the sex hormone environment in postmenopausal women. The FSH/PRL ratio showed a protective association with all three DBP types (Table 8, *P* < 0.05) in this study. Although both FSH and PRL individually promote stiffness and endothelial dysfunction, their combined ratio may facilitate a net reduction in DBP. Whether this reflects a functionally "rebalanced" state warrants investigation.

### Scientific, clinical implications and limitations

This work challenges a prevailing paradigm in postmenopausal hypertension research, the isolated examination of single hormones, and presents a more integrated pathophysiological view. Our approach is grounded in the standardized STRAW + 10 staging system for reproductive aging, which provided the essential framework to systematically investigate hypertension through the lens of ovarian decline-driven sex hormone imbalance [25]. A key finding is that the sex hormone environment exhibits stage-dependent associations with multiple BP parameters. This establishes a novel conceptual link between the dynamics of reproductive aging and the dysregulation of BP, offering a fresh perspective for managing hypertension in this population.

These observations translate into a tangible clinical implication: specific hormone ratios hold promise as pragmatic biomarkers in the future. They could inform personalized management by complementing standard antihypertensive therapy. Importantly, our data point to distinct therapeutic windows. In the early post menopause, attention to the T/PRL-E_2_ balance may be key to safeguarding the regulation of nocturnal SBP. Later in the transition, the FSH/T and PRL-E_2_/T ratios may emerge as potential foci for intervention, where monitoring and correction of imbalances might be particularly relevant.

Collectively, our findings move the field toward a stratified management model. By elucidating how specific hormonal signatures correlate with BP at different menopausal durations, we provide a rationale for individualized, stage-adapted strategies. This refined approach, which targets the underlying endocrine milieu, carries significant potential to mitigate HMOD and reduce cardiovascular risk in postmenopausal women.

Regrettably, this study has several limitations. First, this was a cross-sectional study that can only report an association between sex hormone levels and ABP at a specific time point, without establishing causality or a temporal sequence. Second, the study population was recruited from a single center with a limited sample size, potentially introducing selection bias; therefore, caution should be exercised when generalizing the findings to other populations. Finally, the underlying pathophysiological mechanisms were not thoroughly investigated in this study.

## Conclusions

In women with varying durations of menopause, ABP parameters, particularly nocturnal SBP, are significantly correlated with sex hormone imbalances resulting from declining ovarian function. These findings underscore the importance of tailoring hypertension management and preventing HMOD and CVD in postmenopausal women according to menopausal stage. Further research is needed to elucidate the underlying mechanisms and develop targeted interventions.

## Data Availability

All data generated or analyzed during this study are included in this published article.

## Abbreviations

BP: Blood pressure
ABP: Ambulatory blood pressure
ABMP: Ambulatory blood pressure monitoring
CVD: Cardiovascular disease
SBP: Systolic blood pressure
DBP: Diastolic blood pressure
HMOD: Hypertension-mediated organ damage
E_2_: Estradiol
FSH: Follicle-stimulating hormone
LH: Luteinizing hormone
T: Testosterone
PRL: Prolactin
P: Progesterone
HPO: Hypothalamic‒pituitary‒ovarian
BMI: Body mass index
UA: Uric acid
Cr: Creatinine
FPG: Fasting plasma glucose
WBC: White blood cell
RBC: Red blood cell
PLT: Blood platelet
Hb: Hemoglobin
TC: Total cholesterol
TGs: Triglycerides
HDL-C: High-density lipoprotein cholesterol
LDL-C: Low-density lipoprotein cholesterol
AST: Aspartate aminotransferase
ALT: Alanine aminotransferase
BUN: Blood urea nitrogen
RDWSD: Red blood cell distribution width standard deviation
RDWCV: Red blood cell distribution width coefficient of variation
SD: Standard deviation
AFC: Antral follicle count
ET-1: Endothelin-1
PGI_2_: Prostacyclin
